# Phase I/II study of oral etoposide plus GM-CSF as second-line chemotherapy in platinum-pretreated patients with advanced ovarian cancer

**DOI:** 10.1038/sj.bjc.6602427

**Published:** 2005-03-08

**Authors:** M Baur, E Schernhammer, M Gneist, P Sevelda, P Speiser, M Hudec, Ch Dittrich

**Affiliations:** 1 Applied Cancer Research-Institute for Translational Research VIEnna (ACR-ITR VIEnna), Vienna, Austria; 2 Ludwig Boltzmann-Institute for Applied Cancer Research (LBI-ACR VIEnna), Vienna, Austria; 3 3rd Medical Department with Oncology, Kaiser Franz Josef-Spital, Vienna, Austria; 4 Channing Laboratory, Harvard Medical School, Brigham and Women's Hospital, Boston, MA, USA; 5 Department of Gynecology and Obstetrics, Krankenhaus Lainz, Vienna, Austria; 6 Department of Gynecology and Obstetrics, University of Vienna, Vienna, Austria; 7 Department of Statistics, University of Vienna, Vienna, Austria

**Keywords:** etoposide, dose intensity, GM-CSF, advanced ovarian cancer, salvage therapy, platinum pretreatment

## Abstract

The aim of this phase I/II study was to determine the maximum tolerated dose (MTD) and the dose-limiting toxicities of chronic oral etoposide given on days 1–10 followed by rescue with subcutaneous (s.c.) granulocyte-macrophage colony-stimulating factor (GM-CSF) on days 12–19 as second-line chemotherapy in platinum-pretreated patients (pts) with advanced ovarian carcinoma. Cohorts of three to six pts were treated with doses of oral etoposide from 750 mg m^−2^ cycle^−1^ escalated to 1250 mg m^−2^ cycle^−1^ over 10 days, every 3 weeks. Subcutanous GM-CSF, 400 *μ*g once daily, days 12–19, was added if dose-limiting granulocytopenia was encountered. In total, 18 pts with a median Karnofsky index of 80% (range, 70–100%) and a median time elapsed since the last platinum dose of 10 months (range, 1–24 months), 30% of whom showed visceral metastases, were treated at four dose levels (DLs) of oral etoposide on days 1–10 of each cycle as follows: DL 1, 750 mg m^−2^ cycle^−1^, without GM-CSF, three pts; DL 2, 1000 mg m^−2^ cycle^−1^, without GM-CSF, three pts; DL 3, 1000 mg m^−2^ cycle^−1^, with GM-CSF, six pts; and DL 4, 1250 mg m^−2^ cycle^−1^, with GM-CSF, six pts. All pts were assessable for toxicity and 16 pts for response. Dose-limiting toxicity (DLT) was reached at DL 4 by three of six pts, showing World Health Organization (WHO) toxicity grade 4. One patient died from gram-negative sepsis associated with granulocytopenia grade 4. Two more pts developed uncomplicated granulocytopenia grade 4. Thus, we recommend that DL 3 can be used for further phase II evaluation (i.e. oral etoposide 1000 mg m^−2^ cycle^−1^, days 1–10, followed by s.c. GM-CSF 400 *μ*g, days 12–19). The clinical complete or partial responses in each patient cohort were: DL 1, one of three pts; DL 2, one of three pts; DL 3, three of five pts; and DL 4, two of five pts. In conclusion, in this phase I/II study, we defined the MTD and the dose recommended for the therapy with oral etoposide given over 10 days followed by s.c. GM-CSF in platinum-pretreated patients with advanced ovarian cancer. Our data demonstrate encouraging activity of this regimen and strongly support its further investigation in a phase II study.

Optimal treatment for patients with resistant ovarian cancer has not been standardised to date. The main goals of salvage therapy in these patients are maximisation of their disease-free survival, performance status, and quality of life, depending on the patient's cumulative toxicities due to previous chemotherapy (Salom *et al*, 2002). A variety of treatment options are available for resistant ovarian cancer, including topotecan, pegylated liposomal doxorubicin, gemcitabine, and etoposide.

Until today, etoposide has remained the only substance available as an oral formulation, allowing for outpatient treatment, thereby markedly increasing the convenience for the patient. Etoposide is a cell-cycle, late S- or early G2-phase-specific cytotoxic agent that inhibits topoisomerase II, which is responsible for the cytotoxic activity of etoposide. Inhibition of topoisomerase II is rapidly reversible when plasma levels of etoposide decline (Greco *et al*, 1991). Therefore, it was hypothesised that chronic oral administration of etoposide may result in significant killing of tumour cells. Clinical data have clearly demonstrated the superiority of the delivery of etoposide in a multiple-day schedule compared with delivery of a single dose every 3–4 weeks (Slevin *et al*, 1989).

In ovarian carcinoma, various schedules of peroral administration of etoposide exist that range from 3 to 21 consecutive days of application per cycle, with single daily doses ranging from 25 to 200 mg m^−2^ and with treatment cycles lasting 2–4 weeks. These schedules result in intended dose intensities ranging from 131 to 283 mg m^−2^ week^−1^ (Maskens *et al*, 1981; Hillcoat *et al*, 1985; Hansen *et al*, 1990; Garrow *et al*, 1992; Markman *et al*, 1992; Marzola *et al*, 1993; De Wit *et al*, 1994; Hoskins and Swenerton, 1994; Seymour *et al*, 1994; Kavanagh *et al*, 1995; Yasumizu and Kato, 1995; Kuhn *et al*, 1996; Tuxen *et al*, 1997; Rose *et al*, 1998; Alici *et al*, 2003). However, the optimal schedule of chronic oral administration of etoposide is still to be defined.

Granulocyte-macrophage colony-stimulating factor (GM-CSF) given subcutaneously (s.c.) is known to reduce the incidence and duration of severe granulocytopenia and to enhance the dose intensity of cytotoxic therapy by stimulating the proliferation and differentiation of haematopoietic progenitor cells as well as the functional activity of effector cells (Font *et al*, 1999).

In this study, we administered GM-CSF s.c. after peroral treatment with etoposide to overcome the dose-limiting myelotoxicity of etoposide and to increase the dose intensity per treatment cycle, thereby increasing the response rate, which ultimately would lead to a prolonged progression-free interval and a shorter time to response.

The aim of this phase I/II study was to determine the maximum tolerated dose (MTD) of oral etoposide given on days 1–10 followed by s.c. GM-CSF on days 12–19. These data could serve as the basis for recommendations for future phase II trials in patients with recurring ovarian cancer and/or patients with ovarian cancer refractory to previous platinum-based treatment.

## PATIENTS AND METHODS

### Eligibility criteria

Women between the ages of 18 and less than 75 years with histologically proven epithelial ovarian cancer and measurable progressive disease or relapse were eligible for entry into this phase I/II study. Patients must have received only one previous chemotherapeutic regimen containing either cisplatin or carboplatin; previous additional treatment with a taxane also was permitted. Further eligibility criteria included adequate bone marrow function (platelets>100 000 *μ*l^−1^, haemoglobin>10 g dl^−1^,leucocytes>3.500 *μ*l^−1^), normal renal function (serum creatinine<1.3 mg dl^−1^ and/or creatinine clearance>60 ml min^−1^), normal liver function (transaminases <2 × the normal value, bilirubin <1.5 g dl^−1^), Karnofsky performance status ⩾70%, and freedom from other malignancies for 5 years or more (except successfully treated basal cell and squamous cell carcinoma of the skin or carcinoma *in situ* of the cervix). Patients were excluded if their expected survival was less than 3 months, had an uncontrolled systemic infection, had received an investigational drug within the last 30 days, had received chemotherapy within 30 days of the first cycle of etoposide, or had been pretreated with etoposide. Written informed consent was obtained from all patients. The study was carried out after having received approval of the hospital's ethics committee.

### Treatment plan

Oral etoposide (Vepesid®) 100 mg capsules were given on days 1–10 of each treatment cycle in two daily doses, divided equally, over the 10-day period until the projected cumulative dose per cycle was reached. GM-CSF 400 *μ*g (Leukomax®) was administered s.c. over eight consecutive days per cycle (days 12–19), preferably in the evening.

Cycles were repeated every 3 weeks. The duration of treatment was at least three cycles per patient, with an evaluation of the first response planned after this period, and up to six cycles per patient.

### Dose-escalation algorithm

A dose level (DL) was defined as a cohort of three patients who received at least one cycle of the same DL of oral etoposide. The projected DLs of oral etoposide started with 750 mg m^−2^ cycle^−1^ (without GM-CSF) and were increased in a stepwise manner by 250 mg m^−2^ cycle^−1^. At any given DL, an increase of oral etoposide to the next DL was projected if none of the three patients showed a dose-limiting toxicity (DLT), defined as grade 4 (G4) haematotoxicity and/or G3 or G4 nonhaematological toxicity during the previous therapy cycle. If a DLT occurred in at least one of three patients, three additional patients were treated with this dose level. Maximum tolerated dose was reached when two of three patients or two or more of six patients showed G4 haematotoxicity and/or G3 or G4 nonhaematological toxicity. The DL at which fewer than two of six patients showed a DLT represents the dose recommended for further treatment. No individual intrapatient dose escalation was planned.

Granulocyte-macrophage colony-stimulating factor 400 *μ*g s.c. once daily on days 12–19 was added for all consecutive cycles in patients who had experienced G4 granulocytopenia and, at all further DLs once dose-limiting, that is, World Health Organization (WHO) G4 granulocytopenia, had been encountered. Antiemetic prophylaxis consisted of ondansetron 8 mg b.i.d. and, if necessary, the addition of dexamethasone t.i.d. 8 mg perorally.

Termination of treatment after the completion of six therapy cycles was planned. However, in cases of disease progression, treatment was terminated after the third treatment course, the time when patients underwent their first scheduled treatment evaluation. Furthermore, treatment could be stopped at any time in case of unacceptable toxicity or at the request of the patient.

In cases of G4 granulocytopenia or G4 thrombocytopenia, the protocol required a dose reduction to the previous DL for all further treatment. If the patients’ granulocytes and/or thrombocytes did not meet entry criteria at the scheduled retreatment, a delay in treatment of up to 3 weeks was permitted.

### Pretreatment, response and follow-up evaluation

Within 21 days before the first dose of oral etoposide, baseline disease was documented by radiologic imaging (chest X-ray, computed tomography). Within 1 week before application of oral etoposide, all patients underwent a physical examination, including laboratory analyses (complete blood count, serum chemistry, CA-125 analysis, urine analysis) and assessment of their medical history. A physical examination, serum chemistries, and CA-125 analyses were repeated at each cycle, and a complete blood cell count was obtained weekly. Radiologic reassessment (using the same methodology used at baseline) was performed after the third and sixth treatment cycles.

The evaluation of a patient's response to oral etoposide was based on WHO criteria (UICC, 1987). Patients had to have received a minimum of three cycles of therapy to be eligible for the evaluation of response. If there was clinical indication for early progression of disease, however, an evaluation was performed earlier. Assessment of serologic response was performed by serial CA-125 measurements, defining a decrease of the CA-125 baseline value of at least 50% as a clinical response.

For the definition of platinum responsiveness, patients were considered truly platinum resistant if they relapsed within 6 months of completion of their first-line treatment with platinum-containing therapy or showed disease progression during first-line treatment with platinum-containing therapy.

All patients who had received at least one cycle of oral etoposide were included in the safety analyses. Patient monitoring and scoring of toxicity were performed weekly according to the WHO Toxicity Criteria (WHO, 1979). For determination of the DLT, the occurrence of WHO G4 granulocytopenia or thrombocytopenia or WHO G3 or G4 nonhaematological toxicities were assessed and considered.

### Statistical analysis

Time to progression (i.e. time between the start of treatment and the first objective evidence of tumour progression, or the time to censoring) was estimated by the Kaplan–Meier method. Since previous trials did not specify body surface areas, we based comparisons of dose intensities on a standard of 1.75 m^2^ body surface area, which corresponded to a woman with an average weight of 68 kg and an average height of 165 cm.

In calculations that involved treatment duration, 3 weeks was considered 100%, with 1 week corresponding to 33.3%.

In the analyses of the influence of a specific line of oral etoposide on response rate of oral etoposide (see Table 7), we compared the response rates of patients treated in first-line to response rates of patients treated in second-line to response rates of patients treated in third-line, and to response rates of patients treated in fifth-line treatment. Thereby, the group termed ‘third-line’ therapy consisted of studies administering oral etoposide in mixed lines (either third-line, or second/third- or fourth-line, or third/fourth-line or second/third-line treatment).

## RESULTS

A total of 18 patients were entered into this phase I/II study. Their characteristics are shown in Table 1. In accordance with protocol requirements, all patients received first-line pretreatment with a platinum-based regimen and 30% also received a taxane.

### Dose escalation

Patients were entered at four DLs (Table 2), escalating the dose of oral etoposide from 750 mg m^−2^ cycle^−1^ at DL 1 to 1250 mg m^−2^ cycle^−1^ at DL 4. We enrolled three patients at DL 1(750 mg m^−2^ cycle^−1^ without GM-CSF), three patients at DL 2 (1000 mg m^−2^ cycle^−1^ without GM-CSF), six at DL 3 (1000 mg m^−2^ cycle^−1^ with GM-CSF), and six at DL 4 (1250 mg m^−2^ cycle^−1^ with GM-CSF). No DLT was observed in the first three patients treated at DL 1. One of three patients treated at DL 2 experienced WHO G4 granulocytopenia. In conformity with the protocol, we therefore added GM-CSF to the consecutive DL 3. At DL 3, none of three patients showed haematological toxicity G4 or any other nonhaematological toxicity G3/G4.Thus, we increased the dose of oral etoposide to 1250 mg m^−2^ cyle^−1^ in combination with GM-CSF. One patient of three treated at DL 4 developed granulocytopenia G4 and an associated fatal gram-negative sepsis. Consequently, three additional patients were entered at DL 4, totaling six patients at this DL. One of these additional patients developed granulocytopenia G4 associated with a G3 infection including fever and G2 mucositis, necessitating hospitalisation and treatment with broad-spectrum antibiotics. The patient's recovery was uncomplicated, and treatment could be resumed following a delay of only 1 week and with a reduced dose of oral etoposide. A third patient at DL 4 developed G4 granulocytopenia and had to be given antibiotics on the ward in response to G2 phlebitis. Overall, at DL 4, three of six patients experienced G4 granulocytopenia that demanded antibiotic treatment on the ward and prohibited a further escalation in dose. Subsequently, three additional patients were entered at DL 3. Overall, only one of six patients at DL 3 developed G4 granulocytopenia, which was associated with fungal esophagitis and necessitated antimicrobial treatment on the ward. We conclude that, for oral etoposide, the DL 3 dosage of 1000 mg m^−2^ cycle^−1^ on days 1–10 plus GM-CSF s.c. 400 *μ*g daily on days 12–19, repeated every 3 weeks, should be recommended for further phase II studies.

### Toxicities per patient over all cycles

Haematological and nonhaematological toxicities over all the cycles administered to the 18 patients who entered our study are summarised in Tables 3 and 4. At DL 3, that is, at the dose we recommend for phase II studies, we observed G3 or G4 granulocytopenia in three of six patients. The median granulocyte nadirs during the first cycles were 1600 *μ*l^−1^ at DL 1, 600 *μ*l^−1^ at DL 2, 800 *μ*l^−1^ at DL 3, and 300 *μ*l^−1^ at DL 4. In contrast, the median nadirs of granulocytes from cycles 2–6 were 1900 *μ*l^−1^ at DL 1, 1150 *μ*l^−1^ at DL 2, 1200 *μ*l^−1^ at DL 3, and 2200 *μ*l^−1^ at DL 4. If GM-CSF was administered, recovery from granulocytopenia occurred 6 days earlier, that is, after a median of 14 days (range, 6–29 days), whereas granulocytopenia occurred after a median of 20 days (range, 5–33 days) without the addition of GM-CSF. Overall, no cumulative toxicity was observed. Owing to an allergy to GM-CSF in one patient, granulocyte colony-stimulating factor was used for her further treatment. One patient with G4 thrombocytopenia required a one-time platelet transfusion. Severe nonhaematological toxicity was restricted to colitis, which presented with G3 intensity in one patient and with G4 intensity accompanied by eosinophilic infiltration in another patient. All other toxicities were negligible and manageable.

### Dose-intensity analysis

A total of 93 cycles (median, 6; range, 1–6) were administered in 18 patients. A mean of 98% (range, 83–100%) of the planned dose of oral etoposide could be administered, with a mean treatment duration of 116% (range, 100–140%; 100% corresponding to 3 weeks). In 12 of 18 patients, the duration of treatment had to be extended because of granulocytopenia in nine patients (four patients experienced a delay of 1 week, one patient for 2 weeks, and one for 3 weeks, and in three patients for an overall of 6 weeks each), G2 phlebitis in one patient, G2 mycotic esophageal infection in another patient, and G4 eosinophilic colitis in one other patient. A median effective dose intensity of 234 mg m^−2^ week^−1^ at DL 1, of 238 mg m^−2^ week^−1^ at DL 2, of 272 mg m^−2^ week^−1^ at DL 3, and of 417 mg m^−2^ week^−1^ at DL 4 was reached. Overall, an effective median dose intensity of 292 mg m^−2^ week^−1^ for all DLs was achieved.

### Response to treatment and progression-free interval

In total, 16 of 18 patients were evaluable for response. One patient died after the first cycle at DL 4 of gram-negative sepsis associated with G4 granulocytopenia. In another patient, treatment was stopped at the request of the patient after the second treatment cycle at DL 3, and could therefore not be evaluated for response. Clinical complete or partial responses in each patient cohort were observed at DL 1 in one of three patients, at DL 2 in one of three patients, at DL 3 in three of five patients, and at DL 4 in two of five patients. Overall, four patients reached a complete response, three patients reached a partial response, seven patients had no change, and two patients showed disease progression. Seven (44%) patients of 16 patients objectively responded to oral etoposide as second-line therapy. The two patients with tumour progression belonged to the patient cohorts of DL 2 and DL 4.

Six of nine platinum-sensitive patients responded to etoposide as compared with only one of seven platinum-resistant patients (Table 5).

Two of three patients, whose response to pretreatment with a taxane was evaluable, responded to oral etoposide as second-line therapy, whereas five of 12 patients without taxane pretreatment did so. When the evaluation of tumour response was based on the WHO tumour response criteria, the median time to response was 2 months and decreased to 1.5 months when response criteria included levels of serological CA-125 tumour marker.

The median progression-free interval for all patients was 7 months (range, 1+ to 9 months).

In accordance with the protocol, treatment was discontinued in seven patients after they had completed six treatment cycles without disease progression, in eight patients because of tumour progression, in two patients by request of the patient, and in one patient because of death due to sepsis.

## DISCUSSION

In this study, we achieved an increase in dose intensity of etoposide by using GM-CSF, which advanced by 6 days, the granulocyte nadir following etoposide.

In previous trials, several groups (Seymour *et al*, 1994; Kavanagh *et al*, 1995; Tuxen *et al*, 1997; Rose *et al*, 1998) succeeded in intensifying the dose of etoposide administered without severe haematotoxicity by escalating stipulated doses on an individual basis. In contrast, Garrow *et al* (1992) and De Wit *et al* (1994), who did not make such intraindividual increases in etoposide doses, achieved only the lower median dose intensities of etoposide. We did not design our study to consider individual intrapatient escalation of dose. However, with the use of GM-CSF at DL 3, we achieved the same high dose intensity as did Rose *et al* (1998), Tuxen *et al* (1997), Seymour *et al* (1994), and Kavanagh *et al* (1995). It remains to be determined whether further increases in dose intensity could be reached in patients without severe haematoxicity if doses were escalated on an individual basis, in addition to using GM-CSF. However, it is not clear whether still higher dose intensities are of clinical relevance, particularly since previous studies do not support a clear relationship between effective dose intensities and response rates (Table 6) (Garrow *et al*, 1992; De Wit *et al*, 1994; Seymour *et al*, 1994; Kavanagh *et al*, 1995; Tuxen *et al*, 1997; Rose *et al*, 1998).

When comparing our dose recommendation with that of other phase I trials, smaller total doses of etoposide can be administered in schedules with treatment durations of 1–5 days compared to longer lasting treatments. Data from a randomised trial by Clark *et al* (1994), comparing a schedule of 1–5 days to a schedule of 1–8 days, support these findings (Nissen *et al*, 1976; Lau *et al*, 1979; Ogawa *et al*, 1983; Kimura *et al*, 1985). Furthermore, two other phase I trials that explored schedules extended to 21 days recommended a dosage of 50–75 mg m^−2^ day^−1^, which cumulatively corresponds to our total dose of 1000 mg m^−2^ per cycle (Hainsworth *et al*, 1989; Noda *et al*, 1994).

The median response rate in our study was 44%, compared with median response rates of 26% (range, 0–43%) of other second-line treatment trials in patients with ovarian cancer (Maskens *et al*, 1981; Hoskins and Swenerton, 1994; Yasumizu and Kato, 1995; Kuhn *et al*, 1996; Rose *et al*, 1998). Thus, our treatment regimen can be classified as very effective. Reviewing and comparing the duration of treatment of all second-line oral etoposide regimens in patients with ovarian cancer, we observed a marked difference: One 5-day regimen resulted in a 0% response rate (Maskens *et al*, 1981), whereas 10- to 21-day regimens resulted in response rates between 20 and 45% (Hoskins and Swenerton, 1994; Yasumizu and Kato, 1995; Kuhn *et al*, 1996; Rose *et al*, 1998), lending further support to the superiority of our 10-day design. Another potential explanation for the high efficacy of our regimen could be related to an additional cytotoxic effect of GM-CSF, which has been shown to enhance the number of circulating blood monocytes and their functional cytotoxic impact (Wing *et al*, 1989).

In our study, patients with platinum-sensitive disease responded better than patients with platinum-resistant disease (67 *vs* 14%) (Table 5). Previous studies investigating responsiveness to etoposide in platinum-resistant patients describe a median response rate of 20% (range, 0–27%) (Markman *et al*, 1992; Hoskins and Swenerton, 1994; Kavanagh *et al*, 1995; Kuhn *et al*, 1996; Rose *et al*, 1998; Alici *et al*, 2003).Thus, our findings correspond well with those from other trials, lending further support to a definite, although modest, activity in this cohort of patients.

We observed a 2-month median time to response when we evaluated response by objective measurements of tumour dimension, and a 1.5-month median time when the evaluation also included additional, serological measurements of CA-125. Response that occurs early after the start of therapy is important with regard to symptom palliation and should play a role in the selection of treatment. The primary reason for the early tumoricidal effect we observed could be attributed to the 10-day schedule of our regimen. Clark and Cottier (1992) also demonstrated a time-to-response advantage of a 10-day regimen as compared with a 21-day regimen of prolonged oral etoposide in patients with small-cell lung cancer.

A review of 16 studies performed in 395 evaluable ovarian cancer patients treated with oral etoposide reveals a clear relationship between response to oral etoposide and the sequence of etoposide as first-, second-, third-, fourth-, or fifth-line therapy (Table 7) (Maskens *et al*, 1981; Hillcoat *et al*, 1985; Hansen *et al*, 1990; Garrow *et al*, 1992; Markman *et al*, 1992; Marzola *et al*, 1993; De Wit *et al*, 1994; Hoskins and Swenerton, 1994; Seymour *et al*, 1994; Kavanagh *et al*, 1995; Yasumizu and Kato, 1995; Kuhn *et al*, 1996; Tuxen *et al*, 1997; Rose *et al*, 1998; Alici *et al*, 2003; our study). Studies describe reasonable response rates to second- and third-line therapy. Furthermore, a lack of crossresistance between taxanes and oral etoposide was found in our study, which was confirmed by Rose *et al* (1998), Alici *et al* (2003), Hoskins and Swenerton (1994), but not by Kavanagh *et al* (1995).These data support the value and acceptability of oral etoposide as a treatment option in recurring ovarian cancer. The progression-free interval of a median of 7 months in our trial compared favourably with a median of 6 months in the GOG study by Rose *et al* (1998), which, similar to our study, treated patients in second-line therapy with chronic oral etoposide. In contrast, a trial using topotecan or pegylated liposomal doxorubicin as second-line therapy in patients with recurring ovarian carcinoma reported a median progression-free interval of only 4 months (Gordon *et al*, 2001). It is conceivable that the high response rate in our patients had a positive influence on the duration of the progression-free interval among our patients.

Consistant with other studies (Kuhn *et al*, 1996; Rose *et al*, 1998), myelotoxicity was the predominant and DLT in our trial. Yasumizu and Kato (1995), who administered approximately half the dose of oral etoposide administered by Rose *et al* (1998), observed G3 or G4 leucopenia in only 12% of their patients. In our study, one patient with granulocytopenia died due to sepsis; other groups have reported similar adverse events (Maskens *et al*, 1981; Kuhn *et al*, 1996; Rose *et al*, 1998), whereas no patients died in the trial of Yasumizu and Kato (1995). In sum, because of the wide intrapatient and interpatient variability in the bioavailability of oral etoposide, patients who receive oral etoposide must be carefully monitored for myelosuppression and might need to discontinue therapy or have their dose modified if they experience G3 or G4 granulocytopenia (Hande *et al*, 1993). To improve the bioavailability of oral etoposide and to optimise the therapeutic index of etoposide, the prodrug etoposide phosphate can be administered in a prolonged intraveneous low-volume infusion in an ambulant setting as an alternative treatment option to oral etoposide. A further phase I trial consisting of intravenous etoposide phosphate as continuous infusion over 10 days in combination with subcutaneous GM-CSF on days 12–19 is needed to determine the MTD of etoposide phosphate with subcutaneous GM-CSF for recommendations for future phase II trials in patients with recurring ovarian cancer.

The deepest granulocyte nadir occurred in the first cycles, independent of GM-CSF administration or DLs. Moreover, in our study, the positive influence of GM-CSF on the granulocyte counts started only on the second cycle and continued onward. This observation has not been described previously; however, Paccagnella *et al* provided a potential explanation in his investigation of the bone marrow myeloid precursor proliferative activity in patients with lung cancer treated with chemotherapy and GM-CSF. They demonstrated a significantly higher rate of production of the myeloid precursor cells only after the start of the second treatment cycle (Paccagnella *et al*, 1993). Thus, the decision to increase the dose of etoposide at the consecutive DLs in our study was less likely than we expected to be influenced by administration of GM-CSF. Thus, in our trial, an increase of dose intensity achieved by administration of GM-CSF was achieved only from cycle 2 onward. Weiss *et al* (1996) suggested an alternative option for enhancing dose intensities and improving neutrophil nadirs of the first cycle in a randomised phase I study in which GM-CSF was administered 5 days before the start of the first treatment cycle.

In conclusion, we defined the dose of oral etoposide in combination with GM-CSF s.c. recommended for furture phase II trials in patients with advanced ovarian cancer pretreated with platinum. Keeping in mind all the limitations of nonrandomised small clinical trials, we submit that our 44% response rate is superior to the rates achieved by similar trials testing second-line therapy. Moreover, we observed responses in platinum-sensitive and platinum-resistant patients and no crossresistance with taxanes. Thus, in sum, our treatment regimen can be highly recommended for second- and third-line therapy in patients with recurring ovarian cancer.

## Figures and Tables

**Table 1:** Patient characteristics

Number of patients (*n*)	18
Age (years) median (range)	58 (43–72)

Sites of metastases	
One site	9
Two sites	8
Three sites	1

Tumour grade (*n*)	
Well differentiated	1
Moderately differentiated	5
Poorly or undifferentiated	12

Performance status according to Karnofsky (%)	
70	1
80	8
90	4
100	4
NE	1

Time (months) elapsing from last platinum dose to start of oral etoposide	
Median (range)	10 (1–24)

Previous chemotherapy as first-line therapy (no. of patients)	
Platinum based	18
Cyclophosphamide	9
Epidoxorubicin	1
Paclitaxel	5

Response to previous platinum (no. of patients)	
Platinum sensitive	9
Platinum resistant	9

NE=not evaluable.

**Table 2:** Number of patients with World Health Organization grade 4 toxicities, by dose level, at the first treatment cycle

	**Dose level**			
	**1**	**2**	**3**	**4**
Patients treated (number)	3	3	6	6
Oral etoposide (cumulative dose: mg m^−2^ cycle^−1^)	750	1000	1000	1250

GM-CSF	−	−	+	+
Toxicity grade 4				
Granulocytopenia	0	1	1	3
Thrombocytopenia	0	0	0	1
Sepsis (death)	0	0	0	1

GM-CSF, granulocyte-macrophage colony-stimulating factor; −GM-CSF=without GM-CSF; + GM-CSF=with GM-CSF.

**Table 3:** Number of patients (*n*=18) with haematological toxicities (World Health Organization (WHO) grade) over all cycles

	**WHO grade**				
**Toxicity**	**0**	**1**	**2**	**3**	**4**
Leucocytes	0	6	5	4	3
Granulocytes	1	5	3	2	7
Thrombocytes	6	3	2	5	2
Haemoglobin	0	3	10	5	0

**Table 4:** Number of patients (*n*=18) with nonhaematological toxicities (World Health Organization (WHO) grade) over all cycles

	**WHO grade**				
**Toxicity**	**0**	**1**	**2**	**3**	**4**
Nausea/vomiting	5	2	7	4	0
Mucositis	9	6	3	0	0
Diarrhoea	9	2	5	1	1
Constipation	11	1	6	0	0
Infection	9	6	1	1	1
Alopecia	0	0	2	16	0
Skin	12	5	1	0	0
Paraesthesia	17	1	0	0	0
Lethargy	16	2	0	0	0
GOT	16	2	0	0	0
Bilirubin	17	1	0	0	0
Creatinine	17	0	1	0	0
Alkaline phosphatase	16	2	0	0	0

GOT=glutamate oxaloacetic transaminase (U l^−1^), alkaline phosphatase (U l^−1^), bilirubin (mg %^−1^), creatinine (mg %^−1^).

**Table 5:** Response to treatment with etoposide referring to response to previous platinum treatment (*n*=18)

	**CR**	**PR**	**NC**	**PD**	**NE**
Platinum sensitive	4	2	3		
Platinum resistant		1	4	2	2

CR=complete remission; PR=partial remission; NC=no change; PD=progressive disease; NE=not evaluable.

**Table 6:** Overview of clinical studies using oral etoposide in advanced ovarian cancer regarding effective median dose intensity of oral etoposide and response rate

	**Schedule of etoposide**	**Dose escalation of oral etoposide planned**	**Effective median dose intensity (mg m^−2^ week^−1^)**	**Response rate (%)**	**Line of therapy**
Garrow *et al* (1992)	50 mg m^−2^ day^−1^ × 21 days q 4 weeks		175	20	Third
De Wit *et al* (1994)	50 mg m^−2^ day^−1^ × 21 days q 4 weeks		218	16	Second, third
Kavanagh *et al* (1995)	50 mg m^−2^ day^−1^ × 21 days q 4 weeks	Yes	247	0	Fifth
Rose *et al* (1998)	50 mg m^−2^ day^−1^ × 21 days q 4 weeks	Yes	263	31	Second
Seymour *et al* (1994)	100 mg day^−1^ × 7 days q 3 weeks	Yes	267	24	Second, third
Tuxen *et al* (1997)	170 mg m^−2^ day^−1^ × 5 days q 3 weeks	Yes	>283	48	First
Baur *et al*	75 mg m^−2^ day^−1^ × 10 days q 3 weeks	Yes+use of GM-CSF	292	44	Second

**Table 7:** Relationship of response to oral etoposide and sequence of chemotherapy in patients with advanced ovarian cancer

					**Grouped according to line of therapy**		
**Author**	**Line of therapy**	**Patients in each study (*n*)**	**Responding patients (*n*)**	**Response rate (%)**	**Patients (*n*)**	**Responding patients (*n*)**	**Response rate (%)**
Tuxen *et al* (1997)	1	21	10	48			
					21	10	48
Maskens *et al* (1981)	2	18	0	0			
Yasumizu and Kato (1995)	2	14	6	43			
Kuhn *et al* (1996)	2	18	4	22			
Rose *et al* (1998)	2	82	25	31			
Hoskins and Swenerton (1994)	2	31	8	26			
Baur *et al*	2	16	7	44			
					179	50	28
Markman *et al* (1992)	3	18	1	6			
Garrow *et al* (1992)	3	15	3	20			
Alici *et al* (2003)	2,3	22	4	18			
Hansen *et al* (1990)	2,3	20	2	10			
Seymour *et al* (1994)	2,3	41	10	24			
De Wit *et al* (1994)	2,3	25	4	16			
Hillcoat *et al* (1985)	2,3,4	23	5	22			
Marzola *et al* 1993	3,4	17	1	6			
					181	30	17
Kavanagh *et al* (1995)	5	14	0	0			
					14	0	0
